# Control of structural redundancy from the head to trunk in the human upright standing revealed using a data-driven approach

**DOI:** 10.1038/s41598-022-17322-9

**Published:** 2022-08-01

**Authors:** Kazuya Tanaka, Soichiro Fujiki, Tomoaki Atomi, Wataru Takano, Katsuya Hasegawa, Akinori Nagano, Miho Shimizu, Yoriko Atomi

**Affiliations:** 1 Department of Physical Therapy, Faculty of Medical Sciences, Teikyo University of Science, Uenohara-shi, Yamanashi, 409-0193 Japan; 2 Material Health Science, Graduate School of Engineering, Tokyo University of Agriculture and Technology, Koganei-shi, Tokyo, 184-8588 Japan; 3 Faculty of Medicine, Dokkyo Medical University, Mibu-machi, Tochigi, 321-0293 Japan; 4 Department of Physical Therapy, Faculty of Health Sciences, Kyorin University, Mitaka-shi, Tokyo, 181-8612 Japan; 5 Center for Mathematical Modeling and Data Science, Osaka University, Toyonaka-shi, Osaka, 560-8531 Japan; 6 Institute of Space and Astronautical Science/Japan Aerospace Exploration Agency, Sagamihara-shi, Kanagawa, 252-5210 Japan; 7 Faculty of Sport and Health Science, Ritsumeikan University, Kusatsu-shi, Shiga, 525-8577 Japan

**Keywords:** Biomedical engineering, Rehabilitation

## Abstract

The human being dynamically and highly controls the head–trunk with redundant mechanical structures to maintain a stable upright standing position that is inherently unstable. The posture control strategies are also affected by the differences in the conditions of sensory inputs. However, it is unclear how the head–trunk segmental properties are altered to respond to situations that require appropriate changes in standing posture control strategies. We used a data-driven approach to conduct a multipoint measurement of head–trunk sway control in a quiet standing position with differences in the conditions of sensory inputs. Healthy young subjects with 22 accelerometers attached to their backs were evaluated for head–trunk vibration during quiet standing under two conditions: one with open eyes and one with closed eyes. The synchronization of the acceleration and the instantaneous phase was then calculated. The results showed that the synchronization of acceleration and instantaneous phase varied depending on the visual condition, and there were some continuous coherent patterns in each condition. Findings were that the structural redundancy of the head–trunk, which is multi-segmental and has a high mass ratio in the whole body, must be adjusted adaptively according to the conditions to stabilize upright standing in human-specific bipeds.

## Introduction

The human standing posture is based on controlling the body's center of mass within the base of support, which consists of two feet^1,2^. In particular, as humans evolved from quadrupedal walking to upright bipedal walking, the body’s center of mass position has become higher despite the narrow base of support^3^. Furthermore, since the mass of the head and trunk account for 50%–60% of the body’s total mass, and the head and trunk are situated in the upper part of the human body, this structure of upright posture is a factor in making the upright posture unstable^4–7^. Nonetheless, humans manage to skillfully control this physical instability in their standing posture^8–10^.

Moreover, the human body has a mechanically redundant structure since it consists of an extremely large number of tissues, such as the skeleton, muscles, and ligaments. In particular, the trunk has many more joints than the upper and lower extremities. From an anatomical point of view, the trunk is composed of several bones (including vertebrae and ribs) and joints and is continuously supported by soft tissues with various mechanical coefficients, such as ligaments, muscles, and skin. In addition, the spine has a local segmental structure that is classified into cervical, thoracic, and lumbar vertebrae. The thoracic vertebrae form the rib cage with the ribs and sternum, and are highly rigid as a skeletal structure^11^.

The posture control strategies are also affected by differences in the conditions of sensory inputs. In the standing posture, different conditions of sensory inputs, such as eye-opening (EO) and eye-closing (EC), are known to change the control strategies of the head position and center of pressure in posture control tasks^12–14^.

Humans dynamically and highly control the head–trunk, with its redundant mechanical structures, to maintain a stable standing posture^15–17^. It is therefore very important to investigate the role of the head–trunk in human standing posture control^18–20^.

In biomechanics, the link-segment model is one of the most commonly used models for analyzing human standing posture. The link-segment model is often used to perform kinematic analysis of body segmentation by dividing the body into several links and segment parts^21^. In most cases, link-segment models only set up the joints of the extremities as links^22,23^. However, the trunk has multiple anatomical joints, while the range of motion at each joint is less than that of the extremities. It is therefore difficult to divide the joints from the head to the trunk into the link and segment parts of the link-segment model. Previous studies have reported that a three-or-more-segment model is a more appropriate method for analyzing trunk motion^21,24–26^. However, the control of upright standing is usually studied using a single inverted pendulum model or a double pendulum model where the trunk is one rigid body^27^. In addition, few studies have examined how the segmental properties of the head–trunk are altered to respond to unstable conditions and other situations that require appropriate changes in standing posture control strategies.

In order to clarify these points, it is necessary to examine the behavior of redundant structures from the head to the trunk in different stability and standing posture control conditions, using a multipoint, data-driven method instead of a model-based method to explain how segmentation is controlled. However, to date, no such studies have been found. The purpose of this study, therefore, is to examine the segmental response of the head–trunk with a high body mass ratio in the standing posture, and to determine whether the segmental response is altered by changing visual feedback conditions.

This segmental response can be identified by the magnitude and phase information of the vibration characteristics^28–30^. In the field of engineering, vibration characteristics are often used to investigate physical properties^31^.

Although the human body appears to be stationary in the standing posture, it is known that each part of the body vibrates in a certain range^8,26,32^. In the standing posture, the vibration of body parts propagates between the structures while increasing or decreasing^33^. Therefore, the vibration characteristics of the structures are similar at the sites with low segmentation^28,34^. However, when the vibration characteristics of the structures are different, the segmentation is high^28^. Therefore, the similarity of the vibration characteristics can be used to evaluate the segmentation^28–30^.

In the analysis of standing posture, accelerometers with high time resolution and sensitivity have proven to be useful in verifying minute changes in vibration characteristics in posture control^28^. When using accelerometers in this analysis, the magnitude and direction of the instantaneous acceleration are often analyzed^35–38^. In addition, analysis that uses phase information from time series changes in acceleration can detect differences in vibration characteristics even when there is no difference in the magnitude of the vibrations^39,40^. Therefore, the phase information of the vibration characteristics can be analyzed for their synchronicity to detect similar motions in time series^28–30^.

This study used accelerometers to investigate how to control structural redundancy in the head–trunk to maintain a standing posture under two visual feedback conditions: EO and EC.

## Methods

### Subjects

Data were collected from ten healthy adult males, ranging in age from 20 to 22 years old (mean ± SD: 20.9 ± 0.7 years). The average height was 171.2 ± 4.6 cm and average weight was 68.9 ± 6.3 kg. All participants had no history of orthopedic disease. Written informed consent was obtained from each subject. The study protocol was conducted in accordance with the guidelines proposed in the Declaration of Helsinki, and the Ethics Committee approved the study protocol of Teikyo University of Science (Approval No. 20A018).

### Tasks

The task was designed based on the simple and clinically common Romberg test, which assesses upright postural stability in response to the presence or absence of visual inputs. It is well known that humans often heavily rely on vision to maintain a stable control over their upright standing^41^. Each subject was asked to stand barefoot on a flat surface with the insides of both feet touching. Tasks were conducted under the EO and the EC conditions, and the same condition was measured two times consecutively. The order of each condition was randomized. The duration of each session was 20 s and two trials were measured for each subject. A 2-cm diameter circle was presented at the eye level, 2 m away from and in front of each subject, in all conditions, as an index. In the EO condition, measurements were taken while constantly gazing at the index. In the EC condition, the subjects first gazed at the index, then closed their eyes, and after 3 s, the measurement was started.

### Measurements and methods

A small three-axis acceleration sensor module MMA7361LC (Freescale Semiconductor Inc., Japan) was used to measure the vibration characteristics. The specifications of the accelerometer are as follows: Measurement range: ± 58.8 m/sec^2^ (± 6G), sensitivity: 206 mV/G, response frequency: DC to 1500 Hz, noise: 350 μG/√Hz (0.1 Hz to 1000 Hz), module size: 10 mm (length) × 10 mm (width) × 3.56 mm (height), mass: 2 g.

**Figure 1:**
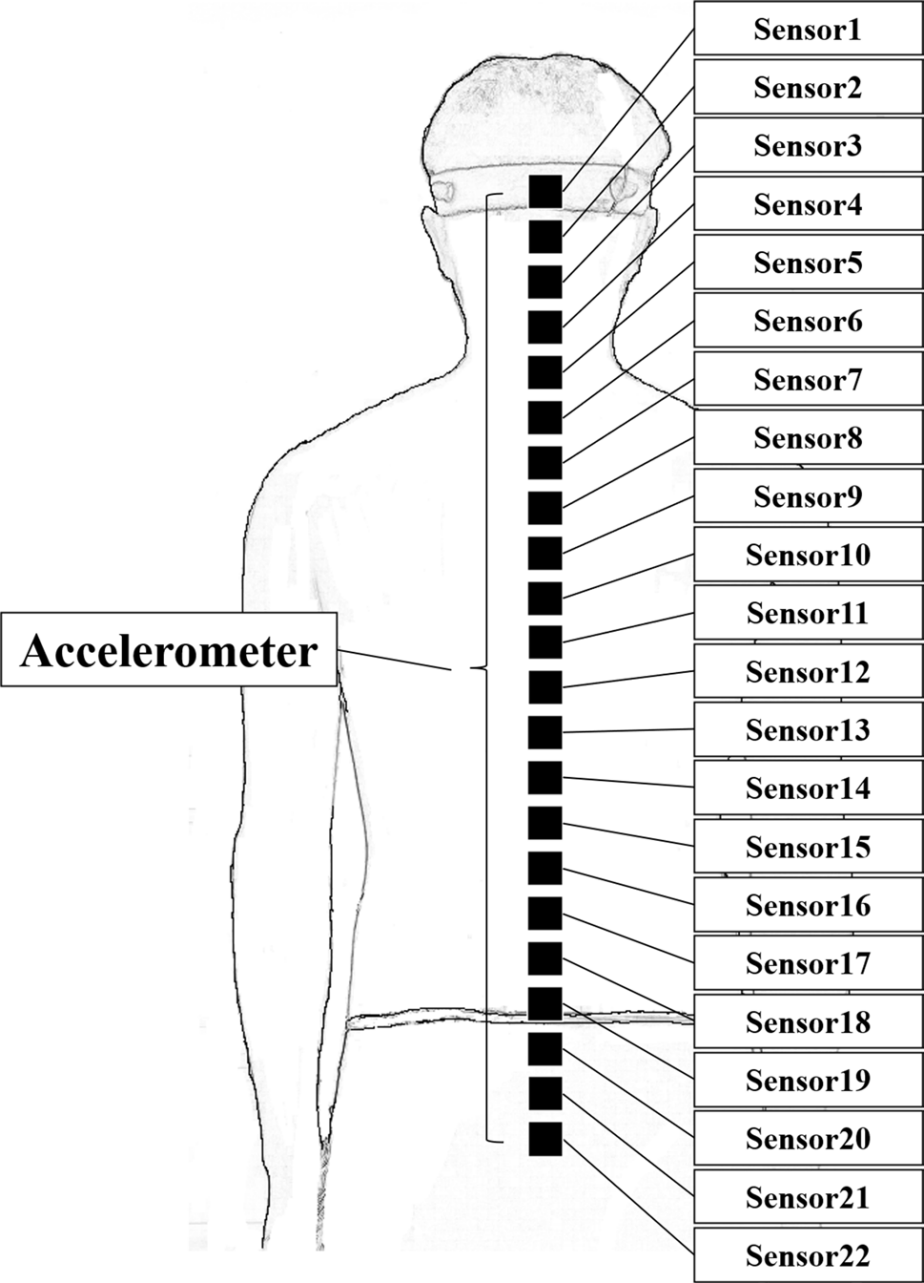
Schematic of 22 accelerometers placement. A triaxial acceleration sensor module was attached at 22 points evenly spaced from the occipital ridge to the sacrum.

We evaluated differences in acceleration characteristics captured by two sensors attached at arbitrary positions. In addition, to perform a data-driven analysis, 22 sensors were evenly attached from the head to pelvis. The sensor-attaching site and data acquisition method for vibration measurement during standing posture control are as follows: A three-axis acceleration sensor module was attached to 22 points on the skin from the head to the sacrum (Fig. 1). These 22 points were equally spaced from the occipital external protuberance to the sacrum. The sampling frequency was 1000 Hz. The analog signal output from the acceleration sensor was inputted to an A/D conversion board (NI USB-6225, National Instruments, USA), and the data was imported to a notebook PC using LabVIEW 2012 (National Instruments). The data obtained from the accelerometer was extracted as acceleration in the three axes of three-directional Euclidean space.

### Data processing

For the analysis, the study used the acceleration in the medial–lateral (ML) and the anterior–posterior (AP) directions after correction by initial posture estimation. The accelerations were low-pass filtered at 20 Hz to remove high-frequency noise^42^. Using the acceleration values after signal processing, similarities in acceleration, and instantaneous phase synchronization were calculated.

### Similarities in acceleration

To examine the acceleration similarity of each measurement point, the correlation coefficients of acceleration between each sensor were calculated for the ML and the AP directions, respectively.

### Instantaneous phase synchronization of acceleration

In the analysis of oscillatory signals such as an electroencephalogram, a measure called phase locking value (PLV) is used to examine the synchronization between measurement points^43–47^. In this study, time-averaged PLV was calculated to investigate the synchronization of the motion occurring at each sensor. First, the Hilbert transform was performed on each time series of acceleration data acquired from sensors in the ML and the AP directions, and then the instantaneous phase $^{ML} \phi_{n} \left( t \right)$ and $^{AP} \phi_{n} \left( t \right)$ was calculated with respect to sampling time *t*. Next, the difference between the instantaneous phases $^{j} \phi_{n} \left( t \right)$ and $^{j} \phi_{m} \left( t \right)$ of the sensors *n* and *m* in the *j* direction at sampling time *t* was notated in the complex number plane, and finally the data was averaged over the total number of samplings times and defined as $^{j} v_{nm}$.

$$ {}_{ }^{j} v_{nm} = \frac{{\mathop \sum \nolimits_{{\text{t}}}^{{\text{T}}} \exp \left\{ {i\left[ {{}_{ }^{j} \phi_{m} \left( t \right) - {}_{ }^{j} \phi_{n} \left( t \right)} \right]} \right\}}}{T} $$

If the absolute values are taken for $^{j} v_{nm}$, it produces the time-averaged PLV, but PLV only expresses the synchronicity in terms of the magnitude of the vectors, not whether they are in-phase or anti-phase.

So,

$$ {}_{ }^{j} \theta_{nm} = \arg \left( {{}_{ }^{j} v_{nm} } \right) $$

and the index of synchronization $^{j} V_{nm}$ was defined as follows:

$$ {}_{ }^{j} V_{nm} = \left| {{}_{ }^{j} v_{nm} } \right|\frac{{\cos {}_{ }^{j} \theta_{nm} }}{{\left| {\cos {}_{ }^{j} \theta_{nm} } \right|}} $$

These indicate that if the value of $^{j} V_{nm}$ is close to 1, the sensor is strongly synchronized in in-phase; if it is close to − 1, the sensor is strongly synchronized in anti-phase; and if it is close to 0, the sensor is weakly synchronized in in-phase or anti-phase. Using these indices, the study investigated the phase synchronization between all the sensors.

### Data analysis

As the similarity of acceleration, the acceleration correlation (AC) of each subject was calculated for each sensor combination, and then the average of AC (AAC) of each sensor combination for each subject was calculated.

As the degree of synchronization of the instantaneous phase at each sensor combination, the average of PLV (APLV) for the ten subjects was calculated after calculating the individual PLV of each subject.

In this study, the following index of AAC and APLV in each sensor combination was used to indicate the degree of segmentation^34,48^: 0.8 or more is “very strong” similarity/synchronization, and the segmentation is clearly reduced in the region. More than 0.6 but less than under 0.8 is “strong” similarity/synchronization, with reduced segmentation. More than 0.4 and less than under 0.6 is “moderate” similarity/synchronization, with a certainly reduced segmentation. Less than under 0.4 is “weak” similarity/synchronization and a clearly high degree of segmentation.

The mean and standard deviation of the acceleration similarity and instantaneous phase synchronization is shown in the heat map.

A two-way repeated measures ANOVA (2 visual feedback conditions × 21 sensor combinations) with Bonferroni post hoc correction was performed for both AC and PLV in each direction at α = 0.05.

MATLAB R2020b (Mathworks Inc., USA) with Signal Processing Toolbox was used to calculate the similarity of acceleration and instantaneous phase synchronization. Statistical analyses were performed using JASP v. 0.16.1 (Univ. of Amsterdam, Netherlands).

## Results

### Similarity of acceleration within subjects

**Figure 2:**
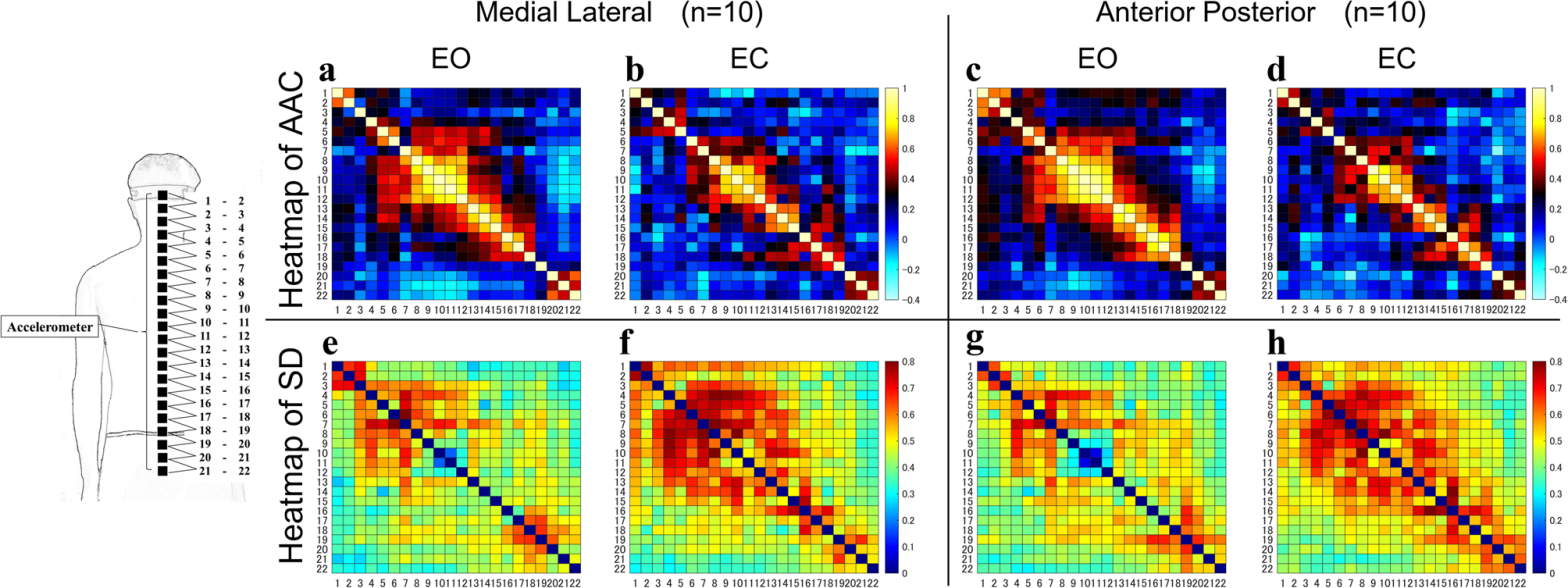
Mean and standard deviation of the similarity of acceleration between each sensor. The heatmaps of AAC (**a–d**) and SD (**e–h**) are shown for each condition (EO and EC). In the heat map of AAC, the left vertical and horizontal axes are the sensor numbers and the right vertical axis is the similarity. In the heat map of SD, the left vertical and horizontal axes are the sensor numbers and the right vertical axis is the standard deviation value. AAC: average acceleration correlation, SD: standard deviation, ML: medial–lateral direction, AP: anterior–posterior direction, EO: eye-opening condition, EC: eye-closing condition.

The following results were obtained for AAC, which indicates the similarity of acceleration of each sensor in each subject (Fig. 2) (Table 1).

**Table 1:** Acceleration similarity between sensors (AAC). The mean acceleration similarity and category between each sensor are shown for each condition. AAC: average acceleration correlation, VS: very strong, S: strong, M: moderate, W: weak, SD: standard deviation, EO: eye-opening condition, EC: eye-closing condition.

Sensorcombination	Medial Lateral (n = 10)						Anterior Posterior (n = 10)					
	EO			EC			EO			EC		
	Mean	SD	Category	Mean	SD	Category	Mean	SD	Category	Mean	SD	Category
1–2	0.60	0.67	S	0.34	0.77	W	0.62	0.64	S	0.47	0.68	M
2–3	0.00	0.71	W	0.20	0.60	W	0.46	0.66	M	0.34	0.61	W
3–4	0.27	0.60	W	0.36	0.53	W	0.28	0.53	W	0.24	0.63	W
4–5	0.43	0.56	M	0.49	0.56	M	0.38	0.63	W	0.37	0.65	W
5–6	0.63	0.45	S	0.09	0.76	W	0.61	0.50	S	0.16	0.78	W
6–7	0.20	0.82	W	0.47	0.75	M	0.38	0.76	W	0.40	0.76	M
7–8	0.67	0.58	S	0.37	0.82	W	0.62	0.62	S	0.34	0.79	W
8–9	0.76	0.48	S	0.66	0.62	S	0.78	0.39	S	0.52	0.73	M
9–10	0.84	0.40	VS	0.73	0.55	S	0.86	0.30	VS	0.77	0.48	S
10–11	0.90	0.17	VS	0.71	0.50	S	0.91	0.08	VS	0.68	0.58	S
11–12	0.80	0.33	VS	0.68	0.53	S	0.83	0.28	VS	0.68	0.55	S
12–13	0.65	0.51	S	0.57	0.61	M	0.61	0.49	S	0.51	0.62	M
13–14	0.72	0.45	S	0.61	0.60	S	0.77	0.40	S	0.64	0.63	S
14–15	0.71	0.40	S	0.67	0.50	S	0.65	0.51	S	0.66	0.51	S
15–16	0.61	0.47	S	0.23	0.75	W	0.72	0.42	S	0.32	0.77	W
16–17	0.70	0.37	S	0.47	0.64	M	0.76	0.41	S	0.54	0.67	M
17–18	0.48	0.63	M	0.49	0.59	M	0.64	0.52	S	0.66	0.54	S
18–19	0.17	0.67	W	0.51	0.47	M	0.38	0.62	W	0.54	0.51	M
19–20	0.16	0.63	W	0.17	0.61	W	0.24	0.67	W	0.02	0.63	W
20–21	0.43	0.43	M	0.42	0.53	M	0.39	0.47	W	0.36	0.56	W
21–22	0.47	0.48	M	0.44	0.51	M	0.41	0.53	M	0.39	0.63	W

In AAC, there were several consecutive clusters of AAC greater than 0.6 or less than 0.4 in both ML and AP directions of the EO and the EC conditions. The AAC between the sensors varied depending on the EO and the EC conditions. The AAC decreased in both ML and AP in the EC condition as compared to the EO condition. The consecutive sensor combinations showing AAC values higher than 0.6 in the ML direction in the EO condition were 1–2, 5–6, and 7–17, whereas those in the EC condition were 8–12 and 13–15. In the AP direction, the consecutive sensor combinations showing AAC values higher than 0.6 were 1–2, 5–6, and 7–18 in the EO condition and 9–12, 13–15, and 17–18 in the EC condition. The consecutive sensor group 9–12 showed AAC values higher than 0.8 in the EO condition in both the ML and the AP directions, although this value was not reached in the EC condition. The consecutive sensor combinations that showed AAC values lower than 0.4 in the ML direction in the EO condition were 2–4, 6–7, and 18–20, whereas those in the EC condition were 1–4, 5–6, 7–8, 15–16, and 19–20. Similarly, in the AP direction, the consecutive sensor combinations showing AAC values lower than 0.4 in the EO condition were 3–5, 6–7, and 18–21, whereas those in the EC condition were 2–6, 7–8, 15–16, and 19–22.

### Instantaneous phase synchronization within subjects

**Figure 3:**
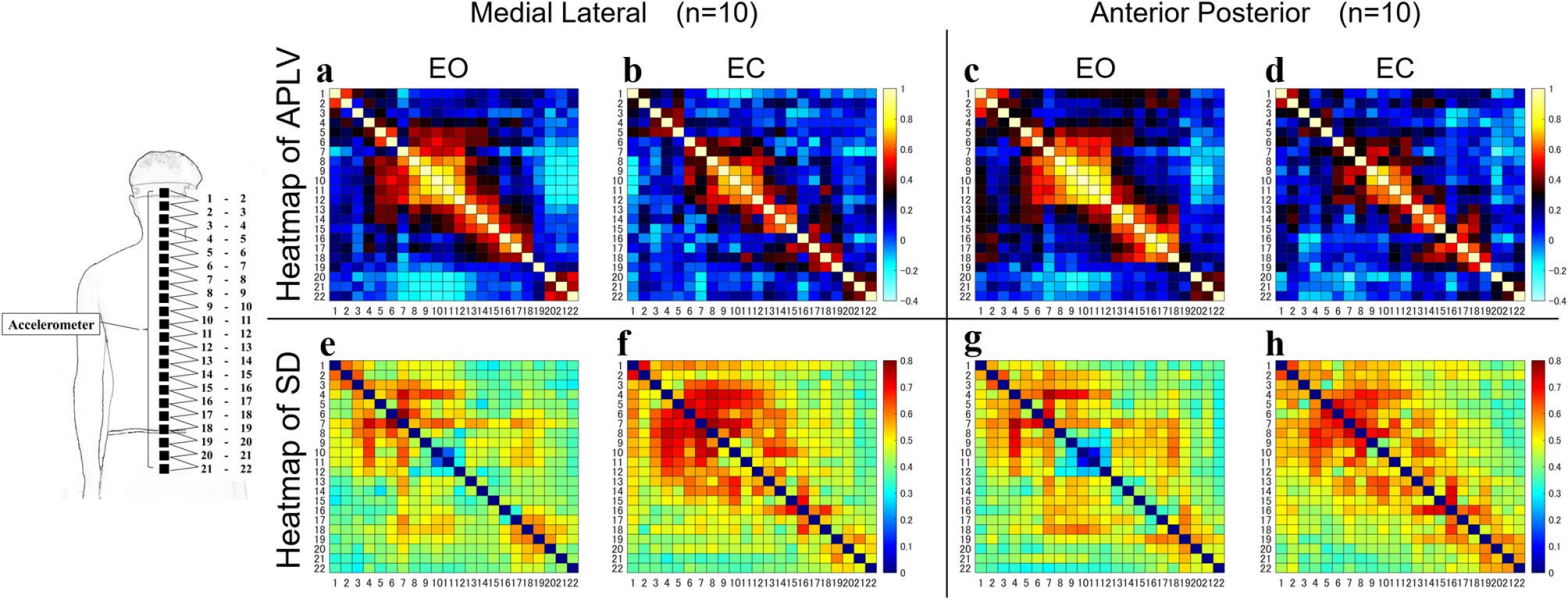
Mean and standard deviation of the instantaneous phase synchronization of acceleration between each sensor. The heat maps of APLV (**a–d**) and SD (**e–h**) are shown for each condition (EO and EC) as phase information in the acceleration time series data. In the heat map of APLV, the left vertical and horizontal axes are the sensor numbers and the right vertical axis is the APLV value. In the heat map of SD, the left vertical and horizontal axes are sensor numbers and the right vertical axis is the standard deviation. APLV: average phase locking value, SD: standard deviation, ML: medial–lateral direction, AP: anterior–posterior direction, EO: eye-opening condition, EC: eye-closing condition.

The APLV, which represents the degree of instantaneous phase synchronization of each sensor in each subject, is shown in Fig. 3 and Table 2.

**Table 2:** Instantaneous phase synchronization between sensors (APLV). The mean instantaneous phase synchronization degree and category between each sensor are shown for each condition. APLV: average phase locking value, VS: very strong, S: strong, M: moderate, W: weak, SD: standard deviation, EO: eye-opening condition, EC: eye-closing condition.

Sensor combination	Medial Lateral (n = 10)						Anterior Posterior (n = 10)					
	EO			EC			EO			EC		
	Mean	SD	Category	Mean	SD	Category	Mean	SD	Category	Mean	SD	Category
1–2	0.56	0.62	M	0.32	0.71	W	0.59	0.57	M	0.46	0.65	M
2–3	0.06	0.63	W	0.18	0.57	W	0.36	0.64	W	0.30	0.58	W
3–4	0.19	0.60	W	0.37	0.49	W	0.29	0.54	W	0.25	0.55	W
4–5	0.38	0.52	W	0.43	0.54	M	0.34	0.60	W	0.33	0.59	W
5–6	0.56	0.43	M	0.10	0.73	W	0.59	0.44	M	0.14	0.73	W
6–7	0.17	0.78	W	0.45	0.72	M	0.40	0.72	M	0.39	0.71	W
7–8	0.64	0.78	S	0.36	0.78	W	0.63	0.59	S	0.30	0.74	W
8–9	0.72	0.44	S	0.64	0.59	S	0.77	0.37	S	0.46	0.69	M
9–10	0.80	0.37	VS	0.70	0.50	S	0.84	0.26	VS	0.73	0.40	S
10–11	0.85	0.17	VS	0.67	0.47	S	0.86	0.10	VS	0.63	0.54	S
11–12	0.73	0.31	S	0.62	0.51	S	0.77	0.26	S	0.64	0.49	S
12–13	0.61	0.42	S	0.51	0.56	M	0.50	0.49	M	0.49	0.55	M
13–14	0.61	0.42	S	0.57	0.55	M	0.72	0.35	S	0.61	0.57	S
14–15	0.60	0.38	S	0.62	0.46	S	0.60	0.43	S	0.60	0.48	S
15–16	0.52	0.46	M	0.17	0.70	W	0.64	0.38	S	0.28	0.72	W
16–17	0.65	0.33	S	0.43	0.60	M	0.74	0.34	S	0.52	0.62	M
17–18	0.44	0.58	M	0.44	0.54	M	0.59	0.50	M	0.59	0.54	M
18–19	0.16	0.60	W	0.49	0.44	M	0.30	0.59	W	0.51	0.47	M
19–20	0.13	0.58	W	0.22	0.56	W	0.18	0.59	W	0.00	0.60	W
20–21	0.40	0.43	M	0.38	0.50	W	0.35	0.47	W	0.28	0.54	W
21–22	0.46	0.42	M	0.41	0.48	M	0.35	0.48	W	0.35	0.58	W

The APLV showed several consecutive clusters of APLV greater than 0.6 or less than 0.4 in both the ML and the AP directions of the EO and the EC conditions. The APLV between sensors varied depending on the EO and the EC conditions. The APLV decreased in both ML and AP in the EC condition as compared to the EO condition. The consecutive sensor combinations showing APLV values higher than 0.6 in the ML direction were 7–15 and 16–17 in the EO condition and 8–12 and 14–15 in the EC condition. In the AP direction, the consecutive sensor combinations showing APLV values higher than 0.6 were 7–12 and 13–17 in the EO condition and 9–12 and 13–15 in the EC condition. In the EO condition, the consecutive sensor combination 9–11 showed APLV values above 0.8 in both the ML and the AP directions, which was no longer the case in the EC conditions. The consecutive sensor combinations showing APLV values lower than 0.4 in the ML direction were 2–5, 6–7, and 18–20 in the EO condition and 1–4, 5–6, 7–8, 15–16, and 19–21 in the EC condition. In the AP direction, the consecutive sensor combinations showing APLV values lower than 0.4 were 2–5 and 18–22 in the EO condition and 2–8, 15–16, and 19–22 in the EC condition.

### Results of two-way repeated measures ANOVA of AC and PLV

The results of two-way repeated measures ANOVA of AC, which indicate the similarity of acceleration, were as follows. There were no significant differences in the main effect of the visual feedback conditions in the ML direction (F (1, 189) = 3.54, *p* = 0.06). There were significant differences in the main effect of sensor combinations in the ML direction (F (20, 189) = 2.33, *p* = 0.002). In post hoc comparisons of sensor combinations, the sensor combination 9–10 was significantly higher than 2–3 (t = 3.83, *p* = 0.04), and 10–11 was significantly higher than 2–3 (t = 3.94, *p* = 0.02). There were significant differences in the main effect of the visual feedback conditions in the AP direction (F (1, 189) = 12.35, *p* < 0.001). In post hoc comparisons of the visual feedback conditions, the EO condition was significantly higher than the EC condition (t = 3.51, *p* < 0.001). There were significant differences in the main effect of sensor combinations in the AP direction (F (20, 189) = 2.13, *p* = 0.005). In post hoc comparisons of sensor combinations, the sensor combination 9–10 was significantly higher than 19–20 (t = 3.89, *p* = 0.03), and 10–11 was significantly higher than 19–20 (t = 3.80, *p* = 0.04).

The results of two-way repeated measures ANOVA of PLV, which indicate the degree of instantaneous phase synchronization, were as follows. There were no significant differences in the main effect of the visual feedback conditions in the ML direction (F (1, 189) = 2.25, *p* = 0.14). There were significant differences in the main effect of sensor combinations in the ML direction (F (20, 189) = 2.34, *p* = 0.002). In post hoc comparisons of sensor combinations, the sensor combination 9–10 was significantly higher than 2–3 (t = 3.78, *p* = 0.04), and 10–11 was significantly higher than 2–3 (t = 3.86, *p* = 0.03). There were significant differences in the main effect of the visual feedback conditions in the AP direction (F (1, 189) = 12.24, *p* < 0.001). In post hoc comparisons of the visual feedback conditions, the EO condition was significantly higher than the EC condition (t = 3.50, *p* < 0.001). There were significant differences in the main effect of sensor combinations in the AP direction (F (20, 189) = 2.37, *p* = 0.001). In post hoc comparisons of sensor combinations, the sensor combination 9–10 was significantly higher than 19–20 (t = 4.30, *p* = 0.006), 10–11 was significantly higher than 19–20 (t = 4.03, *p* = 0.02), and 11–12 was significantly higher than 19–20 (t = 3.80, *p* = 0.04).

In terms of AC and PLV, there were no significant interactions between visual feedback conditions and sensor combinations in both the ML and the AP directions.

## Discussion

This study first focused on APLV and AAC, indicators of the degree of synchronization and similarity between sensors in acceleration waveforms. We determined that the regions with APLV and AAC values less than 0.4 had high segmentation due to “weak” correlations, and regions with values greater than 0.6 had a certain degree of stiffness due to “strong” or higher correlations^34^. Regarding the body regions corresponding to the sensor combination locations, the sensor combination 1–2 corresponds to the head and upper neck, 2–4 to the upper neck, 4–6 to the lower neck, 6–9 to the upper thorax, 9–12 to the middle thorax, 12–15 to the lower thorax, 15–18 to the upper lumbar, 18–21 to the lower lumbar, and 21–22 to the lower lumbar and pelvis.

The APLV and AAC results showed multiple clusters of sensors with high APLV and AAC values on the heat map in the ML and the AP directions for both EO and EC conditions (Figs. 2, 3). In previous studies, it was reported that the phase of instantaneous acceleration tends to be synchronized in models that are close to rigid bodies^30^. Therefore, this study’s measurement sites with high APLV and AAC are sites with low segmentation. The human body’s trunk is a complex structure that is continuously supported by numerous skeletal structures and soft tissues that have various mechanical coefficients, such as ligaments, muscles, and skin^49^, and hence has structural redundancy. Therefore, in controlling the standing posture, the vibration characteristics in the trunk are considered to be continuously changed by the influence of soft tissues, mainly muscles. Our results showed that the sensor groups with APLV and ACC values higher than 0.6 in the ML and the AP directions for all visual feedback conditions corresponded to the thoracic and upper lumbar regions. The spinal column is anatomically divided into cervical, thoracic, lumbar, and sacral vertebrae, which have local segmental structures. The thoracic vertebrae form the rib cage with the ribs and sternum, and are highly rigid as a skeletal structure. The upper lumbar region, which is located between the rigid thoracic and pelvic regions, often showed APLV and AAC values higher than 0.6, although it is a less rigid region in the skeletal structure. It is known that the activity of the erector spinae muscle group in the lumbar back is increased in the standing position^50^. Therefore, it is thought that the stiffness of the lumbar region is increased by the activity of the muscle groups of the lumbar back to supporting the instability in the skeletal structure of the lumbar region.

**Figure 4:**
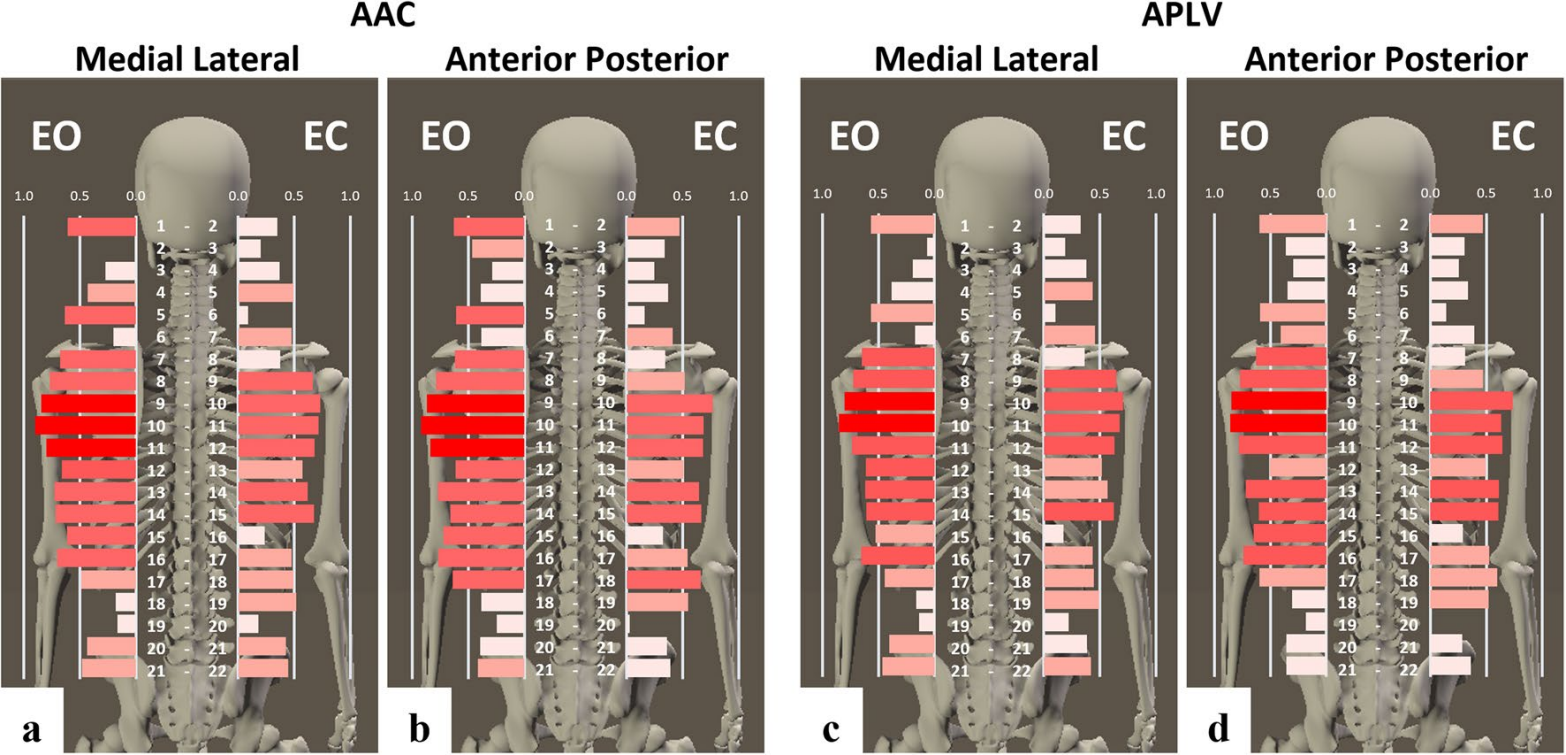
Adaptive segmentation in the head–trunk region with structural redundancy. ACC (**a**) in the medial and lateral directions and ACC (**b**) in the anterior–posterior direction of the adjacent sensors are shown as horizontal bar graphs. APLV (**c**) in the medial and lateral directions and APLV (**d**) in the anterior–posterior direction of the adjacent sensors are shown as horizontal bar graphs. The horizontal axis of the graph shows the AAC and APLV values between the upper and lower sensors. The AAC and APLV values of 21 pairs of sensors from 1 to 22 are shown in order from top to bottom; sensor combinations with high AAC and APLV values are shown in dark red, and sensor combinations with low AAC and APLV values are shown in light red. AAC: average acceleration correlation, APLV: average phase locking value, EO: eye-opening condition, EC: eye-closing condition.

In contrast, APLV and AAC have lower values in regions that show reduced stiffness and segmental movements^30^. Thus, the results suggest that regions with APLV and AAC less than 0.4 are more highly segmented (Fig. 4). The APLV and AAC values of the sensor combinations 3–4 and 19–20 were lower than 0.4 in all visual feedback conditions and directions, which corresponds to the upper neck and lower lumbar region and is consistent with structural anatomical features. These results indicate that the head–trunk is segmented into several parts within the human standing posture to regulate structural redundancy. Taking the whole body into consideration, it is considered a reasonable response to make similar vibration characteristics of large mass at relatively high positions and make some cohesion of redundant structures that could simplify the control variables regulating responding to changes in sensory inputs in standing postures.

Comparison of the APLV and AAC in the EO and the EC conditions revealed that the control strategy for structural redundancy of the head–trunk in the upright posture could change according to the differences in visual feedback conditions (Fig. 4).

High APLV and AAC values indicate an increasing phase synchronization and similarity of acceleration, suggesting an area of increased stiffness and cohesion (Fig. 4). The results of APLV and AAC in the EO condition indicate that the thoracic and upper lumbar regions have a “strong” similarity with phase synchronization of acceleration, and the head and upper neck regions have a “strong” similarity of acceleration. Therefore, it is suggested that the stiffness and cohesion in these regions are increased in the EO condition. On the other hand, APLV and AAC values were reduced in many of these regions in the EC conditions. These results suggest that the redundant response to the EC condition (no visual feedback) as the control of upright posture is regulated by increasing or decreasing the number of apparent head-trunk segments in response to the visual feedback condition.

The low values of APLV and AAC indicate little synchronization and similarity of vibration characteristics, suggesting that they were link sites or high segmentation areas (Fig. 4). Thus, the increase in the number of linked sites indicates that the state is controlled by the increase in the number of segments. This increase in the segments is thought to lead to more joint motion and more input from the sensory receptors around it. The lower values of APLV and AAC in the trunk under the EC condition may respond to the reduction of visual information to increase the input of other sensory systems (e.g., somatosensory) to control the structural redundancy of the trunk. The sensory system is thought to provide the central nervous system with redundant information to maintain body position, and the control system responds to changes caused by a decrease in some sensory information by increasing other sensory inputs and appropriate muscle contractions^51^. This type of neuroscientific mechanism is known as sensory-re-weighting^52–56^. The results suggest that in controlling standing posture, the human body strategically controls the structural redundancy of the head–trunk to respond to changes in the environment and other conditions.

Interestingly, the comparison of APLV and AAC in the EO and the EC conditions showed that the sensor combination 9–11 showed APLV and ACC values higher than 0.8, and the surrounding group of sensors showed characteristically higher values in both the ML and the AP directions in the EO condition, while the range was narrower in the EC condition (Figs. 2, 3). The sensors 9–11 are located in the thoracic region of the skeletal structure. Hence, the response of adding more links in the thoracic region is a common postural control strategy among subjects to compensate for the loss of visual input in the EC condition. In addition, the results of the two-way ANOVA showed that the PLV and AC values in the sensor combination 9–11, corresponding to the middle thorax, showed low segmentation, whereas the upper neck in the ML direction or lower lumbar region in the AP direction showed high segmentation. Therefore, it is suggested that skeletal factors, such as the facet of the cervical and lumbar vertebrae at the link site, are likely influential. This sensor group in the thorax approximates the position of the center of gravity (COG) of the upper body, which is the composite COG of the head, upper arms, and trunk (HAT). In addition, it is believed that human posture and movement are controlled in such a way that the passenger unit, which consists of the HAT, is supported by the locomotor unit, which consists of the lower limbs^57^. The findings of simulation models report that in posture and motion control by human-specific bipeds, setting a virtual control target at a higher position than the COG can dynamically stabilize the instability caused by the high COG^58^.

In this study, a response of additional links was observed in the thoracic region when the loss of visual inputs rendered a change in the control strategy of the upright standing position necessary. Therefore, in order to control the structural redundancy of the trunk in human standing posture, it is considered that one important factor is to make the thoracic region “coherent” to adjust its acceleration. Finally, the overall trend identified using the two-way ANOVA was that the AP direction had significantly higher PLV and AC values in the EO condition than in the EC condition, whereas no significant differences were found between the EO and EC conditions in the ML direction. These results suggest a directional trend in the control of structural redundancy from the head to trunk in response to the loss of visual feedback.

This study showed that the control of structural redundancy of the head, thorax, and lumbar back might be an essential factor in standing posture, but the activity of the muscles connecting the segments has not been examined. In addition, this study’s measurement and analysis methods need to be compared with conditions in which vibration characteristics in the head and trunk may be different (e.g., orthopedic diseases of the spine). In the future, a kinematics study using motion analysis and a neurophysiological study using electromyography will make it possible to develop a more detailed model of trunk control in upright standing. Furthermore, a detailed analysis of how the structural redundancy of the head-trunk is fine-tuned should be conducted by examining the effects of the following experimental conditions: the location of attachments of sensors (e.g., attachments to the spine), the weighting of non-visual sensations such as somatosensory and vestibular sensations, the different visual feedback conditions, and the different foot positions.

The results of this study suggest that the structural redundancy of the head–trunk, which is multi-segmental and has a high mass ratio to the whole body, must be adjusted adaptively according to prevailing conditions in order to control the unstable standing posture within human-specific bipeds. In particular, it was suggested that the instability of the standing posture was controlled by adjusting the acceleration of the thoracic region. It is significant that these findings were obtained by data-driven analysis using time series data.
